# A Vessel-Specific Analysis of Deferred Lesions Using the Instantaneous Wave-Free Ratio and Fractional Flow Reserve

**DOI:** 10.1016/j.jscai.2025.103823

**Published:** 2025-08-19

**Authors:** Karolina Berntorp, Moman A. Mohammad, Sasha Koul, Troels Yndigegn, Ole Fröbert, Anna Myredal, Jonas Persson, David Erlinge, Matthias Götberg

**Affiliations:** a Department of Cardiology, Skåne University Hospital, Lund, Sweden; b Department of Clinical Sciences, Lund University, Lund, Sweden; c Örebro University, Faculty of Health, Department of Cardiology, Sweden; d Department of Clinical Medicine, Faculty of Health, Aarhus University, Aarhus, Denmark; e University of Gothenburg, Institute of Medicine, Department of Molecular and Clinical Medicine, Sweden; f Karolinska Institutet, Department of Clinical Sciences, Danderyd University Hospital, Division of Cardiovascular Medicine, Stockholm, Sweden

**Keywords:** coronary blood flow, left coronary artery, right coronary artery

## Abstract

**Background:**

Physiologically guided revascularization improves clinical outcomes. The cutoff values for deferral with fractional flow reserve (FFR) and instantaneous wave-free ratio (iFR) are the same across all coronary arteries, despite differences in coronary flow patterns. The objective was to compare deferral rates using either FFR or iFR in the right coronary artery (RCA), left anterior descending artery (LAD), and left circumflex artery (LCx), and compare clinical outcomes in deferred lesions in the RCA, LAD, and LCx.

**Methods:**

Right coronary artery, LAD, and LCx lesions in the Swedish Web-System for Enhancement and Development of Evidence-Based Care in Heart Disease Evaluated According to Recommended Therapies registry that were evaluated using either FFR or iFR were included. The composite of major adverse cardiac events (MACE) within 5 years and the individual components of cardiovascular death, noncardiovascular death, myocardial infarction, target segment revascularization, and target vessel revascularization were analyzed.

**Results:**

In total, 33,241 lesions were included in the final analysis (RCA, 17.8%; LAD, 62.3%; and LCx, 19.9%). The median follow-up time was 3.4 years. The median age was 69 years, and 73.5% of patients were men. The deferral rates with iFR were 10.6% higher (*P* < .001) in all coronary arteries combined, 18.7% higher (*P* < .001) in the RCA, 9.5% higher in the LAD (*P* < .001), and 5.3% higher in the LCx (*P* = .007). No significant differences were observed in the MACE rate or its individual components at 5 years between the deferred FFR and iFR groups in any of the investigated vessels.

**Conclusions:**

Instantaneous wave-free ratio demonstrated a higher deferral rate across all coronary arteries than those examined with FFR, which was especially pronounced in the RCA, without any associated increased risk of MACE.

## Introduction

In contrast to the human systemic circulation, coronary circulation is predominantly diastolic.^1^ This reversed circulatory pattern results from compression of the microcirculation by the myocardium during systole, as well as active suction of blood into the coronary vessels during diastole caused by myocardial relaxation.^2^^,^^3^ The left coronary artery (LCA) has a predominantly diastolic perfusion pattern due to the many septal arteries penetrating the myocardium, which are subject to compression and decompression by the myocardium, compared to the right coronary artery (RCA), which has a more evenly distributed perfusion pattern during diastole and systole.^4^, ^5^, ^6^, ^7^

The evaluation of coronary artery stenosis severity by coronary physiology using either the fractional flow reserve (FFR) or instantaneous wave-free ratio (iFR) is recommended in clinical guidelines.^8^^,^^9^ The recommendations do not differ with regard to the coronary vessel of interest, and the cutoff values are the same for all coronary vessels. In the LCA, a decrease in diastolic pressure primarily contributes to the decrease in FFR, whereas in the RCA, the decrease is to a higher extent due to a drop in systolic pressure.^10^

Whereas FFR measures the mean pressure throughout the cardiac cycle under hyperemia induced by adenosine, iFR is a resting index that measures pressure during the wave-free period of diastole.^11^ Despite pivotal studies providing evidence that coronary physiology improves outcomes, further research is needed to evaluate the potential differences in coronary blood flow between the LCA and RCA, their influence on FFR and iFR measurements, and associated clinical outcomes.

The objectives of the study were: (1) to compare deferral rates using either FFR or iFR in the RCA, left anterior descending artery (LAD), and left circumflex artery (LCx); and (2) to evaluate the long-term clinical outcomes of deferred lesions with FFR versus iFR in the RCA, LAD, and LCx in an all-comer real-world population using the Swedish Web-System for Enhancement and Development of Evidence-Based Care in Heart Disease Evaluated According to Recommended Therapies (SWEDEHEART) registry.

## Materials and methods

### Study design and population

Data were collected using the Swedish Coronary Angiography and Angioplasty Registry, which is part of the SWEDEHEART registry, a national quality registry in Sweden that contains data on all patients undergoing coronary angiography, percutaneous coronary intervention (PCI), transcatheter aortic valve replacement, or heart surgery at 30 PCI centers across Sweden. The patients’ unique Swedish personal identification numbers were linked to the Swedish National Population Registry by the Epidemiologic Center of the Swedish National Board of Health and Welfare to obtain information on the vital status of the study population. Data on new myocardial infarction (MI) and unplanned revascularization with coronary artery bypass grafting (CABG) were obtained from the National Patient Register.

**Figure 1:**
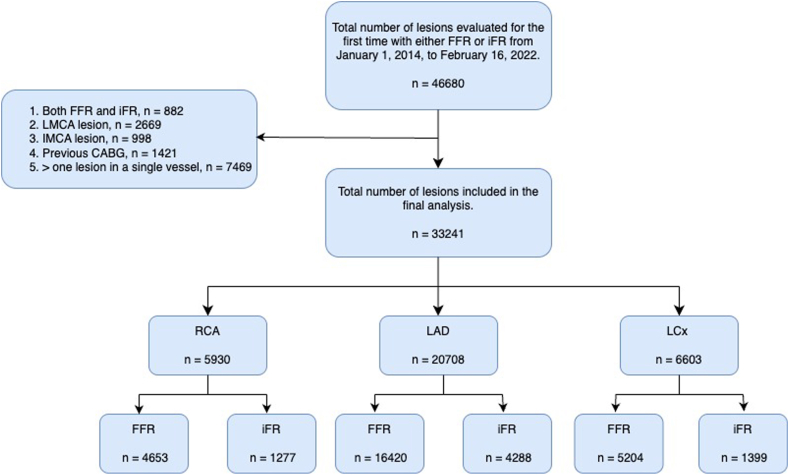
**Flowchart of the study design.** The flowchart illustrates the lesions included and excluded from the study, with numbers representing the lesions remaining at each step. The last 2 steps were stratified according to the vessel investigated and the method of coronary physiology. CABG, coronary artery bypass grafting; FFR, fractional flow reserve; iFR, instantaneous wave-free ratio; IMCA, intermediate coronary artery; LAD, left anterior descending artery; LCx, left circumflex artery; LMCA, left main coronary artery; n, number; RCA, right coronary artery.

All lesions assessed with either FFR or iFR in the Swedish Coronary Angiography and Angioplasty Registry between January 1, 2014, and February 16, 2022, were included. The exclusion criteria were evaluation with both FFR and iFR, the evaluation of more than 1 lesion within a single vessel, left main coronary artery lesions, lesions in the intermediate coronary artery, and previous CABG. Deferral of a lesion was defined as deferral of a lesion evaluated with FFR or iFR, and no planned CABG. A flowchart depicting the study design is shown in Figure 1. The Swedish Ethical Review Authority approved this study (DNR 2023-00201-01).

### Study end points

The deferral rates with FFR and iFR were calculated separately for the RCA, LAD, and LCx. The primary outcome was major adverse cardiac events (MACE) in deferred patients within 5 years for each of the coronary arteries (RCA, LAD, and LCx). MACE was defined as a composite of cardiovascular (CV) death, non-CV death, MI, and unplanned target segment revascularization. Secondary outcomes included individual components of MACE and unplanned target vessel revascularization in the RCA, LAD, and LCx.

### Statistical analysis

Categorical data are presented as counts and percentages, whereas continuous variables are presented as medians and interquartile ranges (IQR). Categorical variables were compared using the χ^2^ test, and continuous variables were compared using the Mann-Whitney U test. Event rates were reported using the Kaplan-Meier analysis at 5 years. Cox regression analysis was used to estimate the hazard ratios (HR) and 95% confidence intervals (CI). If the proportional hazard assumption was not met, a Poisson regression model was applied to calculate the risk ratio (RR) and 95% CI. A multivariable analysis model was used to adjust for the following prespecified variables: age, sex, year of inclusion, smoking status, diabetes mellitus, hypertension, previous history of MI, previous history of PCI, previous history of stroke, chronic heart failure, chronic kidney disease, chronic obstructive pulmonary disease, indication for the procedure, and the segment investigated. Kaplan-Meier curves were constructed to visualize the primary outcome over a 5-year timeframe for each investigated vessel territory. Statistical significance was defined as a 2-sided *P* value <.05. All statistical analyses were performed using STATA SE version 18 (StataCorp LLC).

## Results

### Patient population

**Figure 2:**
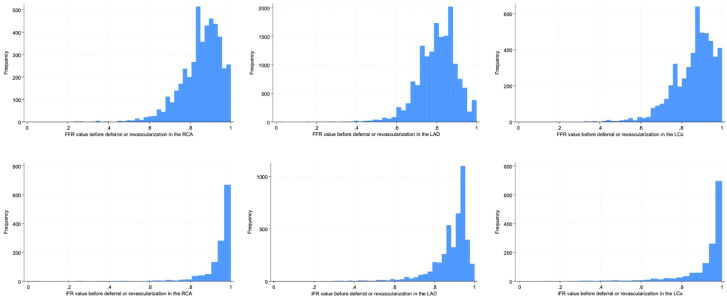
**Histograms of fractional flow reserve (FFR) and instantaneous wave-free ratio (iFR) values.** Illustration of the distribution of FFR and iFR values before deferral or revascularization in each vessel. LAD, left anterior descending artery; LCx, left circumflex artery; RCA, right coronary artery.

A total of 33,241 lesions were included in the final analysis (Figure 1, Central Illustration). Evaluations were performed in the RCA for 5930 (17.8%) lesions, LAD for 20,708 (62.3%) lesions, and LCx for 6603 (19.9%) lesions (Table 1). In the RCA, 78.5% of the lesions were assessed using FFR and 21.5% using iFR. The corresponding numbers for the LAD were 79.3% and 20.7%, and those for the LCx were 78.8% and 21.2%, respectively. The baseline characteristics of the patients are presented in Table 1. The median age of the patients in each group was 69 years, and 73.5% were men. The distribution of FFR and iFR values in each vessel is presented in histograms in Figure 2.

**Central Illustration:**
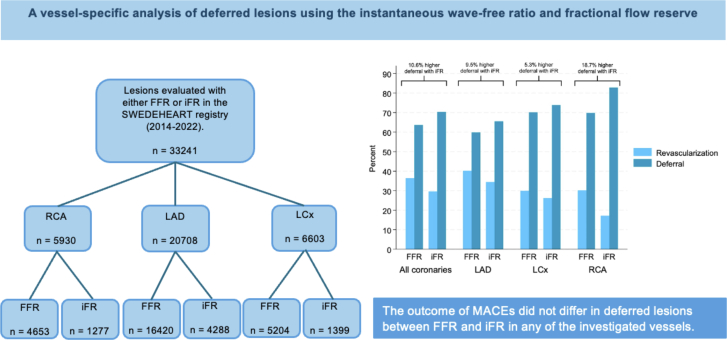
A summary of the study method and outcomes. FFR, fractional flow reserve; iFR, instantaneous wave-free ratio; LAD, left anterior descending artery; LCx, left circumflex artery; MACE, major adverse cardiac event; n, number; RCA, right coronary artery; SWEDEHEART, the Swedish Web-System for Enhancement and Development of Evidence-Based Care in Heart Disease Evaluated According to Recommended Therapies.

**Table 1:** Baseline characteristics.

Variable	RCA					LAD					LCx				
	FFR		iFR		*P* value	FFR		iFR		*P* value	FFR		iFR		*P* value
No. of lesions	4653 (78.5%)		1277 (21.5%)			16,420 (79.3%)		4288 (20.7%)			5204 (78.8%)		1399 (21.2%)		
Segment	1	23.4	1	24.9		6	31.1	6	24.0		11	38.3	11	37.6	.13
	2	42.7	2	36.7		7	53.9	7	56.1		12	30.9	12	31.3	
	3	20.0	3	24.0		8	10.2	8	15.3		13	10.3	13	9.3	
	4	10.3	4	11.1		9	4.1	9	4.1		14	18.8	14	19.1	
	18	3.6	18	3.3		10	0.6	10	0.5		15	1.2	15	2.0	
	19	<1	19	0		20	<1	20	0		16	0.5	16	0.7	
Age, y	69.0 (61.0- 75.0)		69.0 (62.0- 76.0)		.02	69.0 (61.0- 75.0)		69.0 (61.0- 75.0)		.44	69.0 (61.0- 75.0)		69.0 (61.0- 75.0)		.49
Sex					<.001					<.001					.37
Male	3355 (72.1%)		854 (66.9%)			12,060 (73.4%)		3030 (70.7%)			3939 (75.7%)		1075 (76.8%)		
Year of inclusion					<.001					<.001					<.001
2014-2017	2429 (52.2%)		523 (41.0%)			8571 (52.2%)		1695 (39.5%)			2758 (53.0%)		588 (42.0%)		
2018-2022	2224 (47.8%)		754 (59.0%)			7849 (47.8%)		2593 (60.5%)			2446 (47.0%)		811 (58.0%)		
Smoking status					.09					<.001					<.001
Never smoked	1719 (36.9%)		453 (35.5%)			6815 (41.5%)		1710 (39.9%)			2090 (40.2%)		518 (37.0%)		
Ex-smoker	2075 (44.6%)		597 (46.8%)			6906 (42.1%)		1963 (45.8%)			2246 (43.2%)		653 (46.7%)		
Current smoker	714 (15.3%)		202 (15.8%)			2088 (12.7%)		570 (13.3%)			692 (13.3%)		204 (14.6%)		
Unknown	145 (3.1%)		25 (2.0%)			610 (3.7%)		45 (1.0%)			176 (3.4%)		24 (1.7%)		
Medical history															
Diabetes	1269 (27.3%)		353 (27.6%)		.79	4215 (25.7%)		1132 (26.4%)		.33	1455 (28.0%)		364 (26.0%)		.15
Hypertension	3645 (78.3%)		1004 (78.6%)		.83	12,448 (75.8%)		3245 (75.7%)		.86	4085 (78.5%)		1091 (78.0%)		.68
Previous MI	1679 (36.1%)		454 (35.6%)		.73	5029 (30.6%)		1346 (31.4%)		.34	1934 (37.2%)		517 (37.0%)		.89
Previous PCI	2051 (44.1%)		540 (42.3%)		.25	5857 (35.7%)		1525 (35.6%)		.90	2306 (44.3%)		615 (44.0%)		.81
Previous stroke	287 (6.2%)		104 (8.1%)		.01	986 (6.0%)		299 (7.0%)		.02	329 (6.3%)		100 (7.1%)		.27
Heart failure	490 (10.5%)		139 (10.9%)		.72	1553 (9.5%)		395 (9.2%)		.62	540 (10.4%)		139 (9.9%)		.63
COPD	355 (7.6%)		98 (7.7%)		.96	983 (6.0%)		302 (7.0%)		.01	331 (6.4%)		89 (6.4%)		1.00
Renal failure	170 (3.7%)		47 (3.7%)		.96	586 (3.6%)		158 (3.7%)		.72	218 (4.2%)		69 (4.9%)		.23
Indication for the procedure					.04					.001					.34
CCS	1606 (34.5%)		417 (32.7%)			5624 (34.3%)		1451 (33.8%)			1834 (35.2%)		486 (34.7%)		
Unstable angina	743 (16.0%)		225 (17.6%)			2491 (15.2%)		691 (16.1%)			811 (15.6%)		248 (17.7%)		
NSTEMI	828 (17.8%)		191 (15.0%)			2979 (18.1%)		674 (15.7%)			929 (17.9%)		236 (16.9%)		
STEMI	74 (1.6%)		23 (1.8%)			247 (1.5%)		59 (1.4%)			86 (1.7%)		19 (1.4%)		
Other	1402 (30.1%)		421 (33.0%)			5079 (30.9%)		1413 (33.0%)			1544 (29.7%)		410 (29.3%)		
Value of FFR and iFR															
Revascularized lesions	0.77 (0.72-0.84)		0.89 (0.82- 0.96)			0.74 (0.70- 0.78)		0.85 (0.79- 0.88)			0.77 (0.72- 0.84)		0.87 (0.75- 0.95)		–
Deferred lesions	0.90 (0.85- 0.93)		0.97 (0.95- 0.99)			0.85 (0.82- 0.89)		0.93 (0.91- 0.95)			0.90 (0.86- 0.95)		0.98 (0.95- 1.0)		–

Values are n (%) or median (IQR).

CCS, chronic coronary syndrome; COPD, chronic obstructive pulmonary disease; FFR, fractional flow reserve; iFR, instantaneous wave-free ratio; LAD, left anterior descending artery; LCx, left circumflex artery; MI, myocardial infarction; NSTEMI, non–ST-elevation myocardial infarction; PCI, percutaneous coronary intervention; RCA, right coronary artery; STEMI, ST-elevation myocardial infarction.

Significant differences in sex (*P* < .001), year of inclusion (*P* < .001), previous stroke (*P* = .01), and indication for the procedure (*P* = .04) were observed between the FFR and iFR groups in the RCA. In the LAD, significant differences were observed in terms of sex (*P* < .001), year of inclusion (*P* < .001), smoking status (*P* < .001), previous stroke (*P* = .02), chronic obstructive pulmonary disease (*P* = .01), and indications for the procedure (*P* = .001). In the LCx, significant differences were observed only in the year of inclusion (*P* < .001) and smoking status (*P* < .001).

### Outcomes

**Figure 3:**
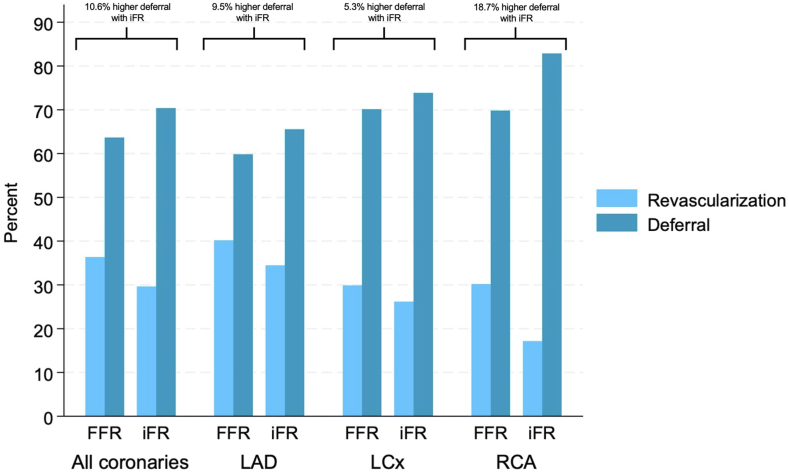
**Deferral rate across all coronary arteries.** The figure illustrates revascularization and deferral rates across all coronary arteries. The increased deferral rates with instantaneous wave-free ratio (iFR) across all arteries are presented as percent higher deferral with iFR compared to fractional flow reserve (FFR). LAD, left anterior descending artery; LCx, left circumflex artery; RCA, right coronary artery.

The median follow-up time was 3.4 years. The deferral rates in the RCA were 72.6% versus 61.0% in the LAD (*P* < .001) and 70.9% in the LCx (*P* = .04). A significant difference in the deferral rates was observed between the LCx and LAD (*P* < .001). The deferral rates in the FFR group versus iFR group (Table 2) were 69.8% versus 82.6% in the RCA (*P* < .001), 59.8% versus 65.5% in the LAD (*P* < .001), and 70.1% versus 73.8% in the LCx (*P* = .007). The deferral rate with iFR was 10.6% higher (*P* < .001) in all coronary arteries combined, 18.7% higher in the RCA, 9.5% higher in the LAD, and 5.3% higher in the LCx (Table 2, Figure 3). After excluding all patients with >1 vessel investigated with either FFR or iFR, and all lesions in the LAD or LCx treated with PCI, the deferral rate was 68.1% with FFR and 81.3% with iFR (*P* < .001).

**Table 2:** Proportion of deferral with FFR and iFR.

	Total	FFR	iFR	FFR vs iFR *P* value^a^
Coronary artery				
All	21,621 (65.0%)	16,720 (63.6%)	4901 (70.4%)	<.001
RCA	4306 (72.6%)	3248 (69.8%)	1058 (82.6%)	<.001
LAD	12,632 (61.0%)	9822 (59.8%)	2810 (65.5%)	<.001
LCx	4683 (70.9%)	3650 (70.1%)	1033 (73.8%)	.007

FFR, fractional flow reserve; iFR, instantaneous wave-free ratio; LAD, left anterior descending artery; LCx, left circumflex artery; RCA, right coronary artery.

a Comparison of the proportion of deferral with FFR vs iFR by coronary vessel with the χ^2^ test.

**Figure 4:**
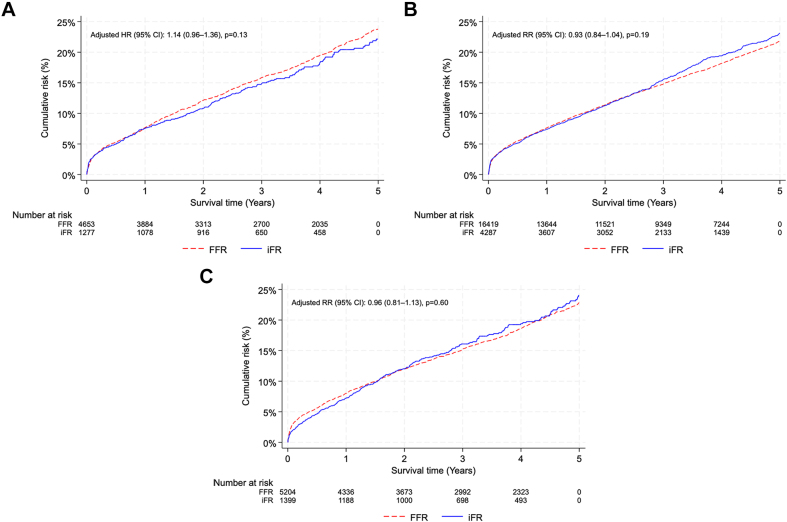
**Kaplan-Meier graphs for the primary outcome**. Kaplan-Meier graphs visualizing the cumulative risk of the primary outcome of major adverse cardiac events over 5 years for the (A) right coronary, (B) left anterior descending, and (C) left circumflex arteries. FFR, fractional flow reserve; HR, hazard ratio; iFR, instantaneous wave-free ratio; RR, risk ratio.

No significant differences were found in the MACE rates at 5 years between the deferred FFR and deferred iFR groups in any of the investigated vessels: RCA (adjusted HR, 1.14; 95% CI, 0.96-1.36; *P* = .13), LAD (adjusted RR, 0.93; 95% CI, 0.84-1.04; *P* = .19), and LCx (adjusted RR, 0.96; 95% CI, 0.81-1.13; *P* = .60) (Table 3). The Kaplan-Meier curves for the primary outcome of MACE over 5 years are presented for each vessel in Figure 4. No significant differences were observed in the secondary outcomes in the RCA, LAD, or LCx lesions. A borderline significant increase was recorded in target segment revascularization with FFR in the RCA (adjusted HR, 1.46; 95% CI, 1.00-2.15; *P* = .05).

**Table 3:** Event rates at 5 years and outcomes^a^ of deferred lesions in separate vessel analysis.

	FFR	iFR	HR (95% CI)	*P* value	Adjusted HR (95% CI)	*P* value
RCA						
MACE	615 (24.6%)	168 (21.8%)	1.14 (0.96-1.35)	.14	1.14 (0.96-1.36)	.13
Myocardial infarction	250 (9.8%)	71 (9.5%)	1.09 (0.84-1.43)	.46	1.08 (0.82-1.40)	.59
CV death	123 (5.8%)	37 (6.1%)	0.99 (0.68-1.42)	.94	1.00 (0.69-1.45)	.98
Non-CV death	168 (8.9%)	55 (9.5%)	0.86 (0.63-1.16)	.32	0.98 (0.72-1.34)	.92
TSR	162 (6.6%)	32 (4.7%)	1.58 (1.08-2.30)	.02	1.46 (1.00-2.15)	.05
TVR	232 (9.5%)	56 (8.3%)	1.29 (0.96-1.72)	.09	1.25 (0.93-1.68)	.14
LAD						
MACE^b^	1657 (22.5%)	453 (22.7%)	1.05 (0.94-1.16)	.40	0.93 (0.84-1.04)	.19
Myocardial infarction	634 (8.6%)	177 (8.5%)	1.00 (0.85-1.18)	.99	0.93 (0.78-1.10)	.39
CV death	305 (5.1%)	91 (5.9%)	0.89 (0.71-1.13)	.34	0.82 (0.65-1.04)	.10
Non-CV death^b^	492 (9.0%)	155 (10.8%)	0.81 (0.68-0.98)	.03	0.84 (0.70-1.00)	.06
TSR^b^	435 (5.6%)	112 (5.6%)	1.11 (0.90-1.37)	.32	0.99 (0.81-1.23)	.96
TVR^b^	733 (9.6%)	195 (9.4%)	1.08 (0.92-1.26)	.37	1.01 (0.87-1.19)	.86
LCx						
MACE^b^	651 (23.6%)	183 (24.4%)	1.0 (0.85-1.19)	.94	0.96 (0.81-1.13)	.60
Myocardial infarction	310 (11.1%)	88 (12.5%)	0.96 (0.76-1.22)	.74	0.97 (0.76-1.23)	.81
CV death	131 (5.9%)	38 (6.5%)	0.90 (0.63-1.30)	.57	0.89 (0.61-1.28)	.52
Non-CV death	178 (8.7%)	51 (9.3%)	0.88 (0.65-1.21)	.44	0.96 (0.70-1.31)	.78
TSR	141 (5.1%)	41 (5.3%)	0.94 (0.66-1.33)	.72	0.93 (0.65-1.32)	.67
TVR	226 (8.3%)	67 (8.4%)	0.92 (0.70-1.20)	.52	0.90 (0.68-1.18)	.43

CV, cardiovascular; FFR, fractional flow reserve; HR, hazard ratio; iFR, instantaneous wave-free ratio; MACE, major adverse cardiac events; TSR, target segment revascularization; TVR, target vessel revascularization.

a Outcomes were adjusted for age, sex, year of inclusion, smoking status, diabetes mellitus, hypertension, previous myocardial infarction, previous percutaneous coronary intervention, previous stroke, heart failure, kidney disease, chronic obstructive pulmonary disease, procedure indication, and lesion segment.

b Did not fulfill the assumption of proportional hazard and are therefore presented as risk ratios calculated with Poisson regression.

## Discussion

In this SWEDEHEART registry study, the main findings were as follows: (1) the deferral rate was significantly higher with iFR than with FFR in the RCA, LAD, and LCx; (2) the difference in deferral rates was especially pronounced in the RCA; and (3) the primary outcome of MACE and its individual components did not differ between FFR and iFR in any of the investigated vessels for up to 5 years.

### Flow pattern in the LCA and RCA

The diastolic flow pattern in the LCA has been investigated and confirmed in several studies.^4^, ^5^, ^6^, ^7^ This is primarily due to the numerous septal arteries that penetrate the myocardium, which are subjected to the large contractile forces of the left ventricle, as well as the pressure gradient across the myocardium. The flow pattern of the RCA is not well understood. Small studies have suggested that the systolic flow is equal to or greater than the diastolic flow.^5^^,^^12^^,^^13^ However, Seligman et al^14^ showed that diastolic predominance is observed in both the RCA and LCA in a more recently published study including 301 patients and 482 simultaneous pressure and flow measurements. They suggest that the clinical interpretation of coronary physiology data should not differ between the RCA and LCA. Another study on FFR, which compared the pressure drop differences between the RCA and LCA, demonstrated that the pressure drop in the LCA is primarily due to a decrease in diastolic pressure, whereas in the RCA, the drop was mainly due to a decrease in systolic pressure.^10^ To the best of our knowledge, no such studies on iFR are available. iFR measurements are performed during the diastolic phase of the cardiac cycle. Thus, whether the potential differences observed in flow patterns during the cardiac cycle between different coronary vessels affect revascularization patterns and clinical outcomes remains unknown. Our study provides deep insights regarding coronary physiology and the potential differences between the RCA and LCA by delving into the clinical outcomes.

Previous randomized studies comparing FFR and iFR have shown that the deferral rate with iFR is significantly higher than that with FFR.^15^^,^^16^ Our real-world large observational study confirmed this, with a higher deferral rate with iFR across all investigated vessels. The potential reasons for this difference can be explained by the discordance between FFR and iFR in the intermediate range of values close to the threshold, as well as better agreement between iFR and coronary flow reserve compared to FFR and coronary flow reserve.^17^, ^18^, ^19^, ^20^ The differences in deferral rate between FFR and iFR seen in our study population are remarkably higher than in the randomized studies. A potential reason could be that it is easier to perform iFR than FFR, which in turn lowers the threshold to perform physiology and subsequently leads to more lesions examined per patient.^16^ This results in more low-grade lesions examined, and consequently, a higher deferral rate. The higher deferral rate with iFR in our deferred study population did not affect the primary outcome of MACE or secondary outcomes in any of the investigated vessels. This suggests that despite the potential differences in coronary flow, the clinical outcomes were not affected.

### Deferral rates depending on the evaluated vessel

We observed a higher deferral rate in the RCA and LCx than in the LAD, irrespective of the modality. In particular, iFR evaluation in the RCA was associated with a higher deferral rate compared with FFR without a difference in MACE, CV death, MI, or revascularization. The difference in deferral rate was not sensitive to PCI performed in the LAD and LCx. Several factors may explain the higher incidence of hemodynamically significant LAD stenosis. The most important factor is that the LAD supplies a larger myocardial mass for a given percentage of stenosis, which results in a higher pressure drop across the stenosis in the LAD compared to the same stenosis in the RCA or LCx. Second, the LAD is more prone to atherosclerosis compared to the RCA, which may lead to a higher pressure drop in the LAD.^21^ Third, hydrostatic differences resulting from height differences between the LAD and the RCA could have played a role in the result of the measurement.^22^^,^^23^ Fourth, pressure decreases proportionally with distance from the ostium in the LAD, even in the absence of stenosis.^24^ The presence of 1 or more of these factors could have contributed to the lower deferral rate observed in the LAD. Additionally, because of the study design, in which the decision to perform revascularization or deferral is at the discretion of the operator, the operator may be more likely to avoid deferral of a stenosis in the LAD than in the RCA or LCx, especially when the FFR or iFR values are of borderline significance. Notably, because coronary physiology is a continuous variable, a decision-making process using cutoff values without integrating clinical context should be avoided outside randomized studies. The observed difference in deferral rate between FFR and iFR in the RCA could reflect differences in coronary flow patterns between the RCA and LAD. Because FFR is measured throughout the cardiac cycle, whereas iFR is measured during diastole, the differences in flow patterns between different vessels could potentially affect FFR and iFR measurements differently. In the previously mentioned study with 482 simultaneous flow and pressure measurements, a diastolic predominance was seen in both the RCA and LCA. However, the magnitude of diastolic dominance was 17% lower for the RCA compared to the LAD. This suggests that despite diastolic predominance in all vessels, the magnitude of diastolic flow could influence the iFR value in stenotic lesions, deferring more lesions. Despite a higher deferral rate using iFR being observed in the RCA in our study, no difference in clinical outcomes was observed, which would likely have been the case if 1 of the indices had missed ischemic lesions.

### Limitations

This study had some important limitations. First, as an observational registry study, the possibility of residual confounding remains despite adjusting for several key confounders. Additionally, the outcome of MI was based on the ICD-10 codes tracked from hospital admissions, which introduces the possibility of misclassification, particularly for less clinically important events. The decision to defer or proceed with revascularization following FFR and iFR measurements was not based on a single cutoff value. Although FFR <0.80 and iFR <0.89 are commonly used as thresholds for revascularization, this study reflects everyday clinical practice, where revascularization decisions are made at the discretion of the operating physician. Comparison of deferral rates and clinical outcomes between vessels, as well as inclusion of patients undergoing both FFR and iFR, was beyond the scope of this study. However, such analyses may be valuable in future research to further explore potential vessel-specific differences between FFR and iFR.

## Conclusion

Evaluation of the hemodynamic significance of a lesion using FFR or iFR showed a higher deferral rate across all coronary arteries with iFR compared to FFR, which was particularly pronounced in the RCA, without any associated increase in the risk of MACE among deferred lesions.
